# Supplementary material for: MetaRibo-Seq measures translation in microbiomes
Source: Nat Commun. 2020 Jun 29;11:3268. doi: 10.1038/s41467-020-17081-z (PMC7324362; doi:10.1038/s41467-020-17081-z)
Supplement: Supplementary file 10 — Supplementary Data 7 [file 41467_2020_17081_MOESM10_ESM.zip › File2/Confidence_High_Taxonomy/46791_out.krona.html]

Javascript must be enabled to view this page.

members
magnitude
magnitudeUnassigned
count
unassigned
taxon
rank

46791\_out

3

superkingdom
3
2

phylum
1239
3

class
186801
3

186802
3
order

family
541000
1

genus
1
1935176

1
1871018
species

SRS147139\_contig\_number\_contig-100\_38486.71056

2
1898207
species

SRS098571\_contig\_number\_contig-100\_35025.173078SRS104636\_contig\_number\_13995
